# The mechanism analysis on the farmers’ motivation of using the quality traceability system based on TAM-ECM model

**DOI:** 10.1038/s41598-023-49795-7

**Published:** 2023-12-14

**Authors:** Wang He, Chunjie Qi, Liang Ding

**Affiliations:** 1 College of Economics and Management, Huazhong Agriculture University, Wuhan, 430070 China; 2 School of International Trade and Economics, Jiangxi University of Finance and Economics, Nanchang, 330013 China; 3 Applied Economic Research Center, Jiangxi Institute of Fashion Technology, Nanchang, 330201 China

**Keywords:** Environmental economics, Psychology and behaviour

## Abstract

The quality traceability system for agricultural product plays an important role in tracing the production history or flow of agricultural products in China. At present, the quality traceability system is facing the problem of limited coverage and promotion. Understanding the continuous usage behavior of users can help solve this problem. This study constructed a model integrating TAM-ECM to analyze the mechanisms affecting the continuous usage intention and behavior of users of the quality traceability system, using PLS analysis method and survey data from 197 users of the GanNan navel orange quality traceability system with a usage time of up to two years. The results show that both satisfaction and usage habits directly promote the continuous usage of the quality traceability system. Lowering transaction costs significantly improves perceived value and satisfaction, and has a greater direct impact on satisfaction. Expectation confirmation level has a positive effect on perceived value, and lowering transaction costs also enhances perceived value. Perceived value、transaction cost, and satisfaction are important factors driving the continuous usage of the quality traceability system by users. The research findings provide a reference basis for the government to develop policies to attract users to use the quality traceability system for agricultural product.

## Introduction

The quality traceability system for agricultural products, refers to the use of information technology, to attach a "special" label to agricultural products. This label includes information such as planting, processing, packaging, logistics, etc. As long as the label is queried, it can trace the handlers of agricultural products in each link. This provides a reliable basis to solve food safety and quality supervision problems, which is regarded as a quality assurance system that records and stores various product-related information throughout the entire process of product supply^1^. Its purpose is to quickly and efficiently trace the source of food safety issues, isolate problematic food from the supply chain, and minimize the risk of harm to public health. In recent years, national laws and regulations strongly support the construction and promotion of agricultural product traceability system platforms in Chin. However, the users did not show strong willingness to participate in agricultural product quality traceability systems^2,3^.

It is our interests to analysis what causes the weak willingness to use the agricultural product quality traceability system? What factors that affect producer user behavior? I.e., the producer's own characteristic variables, such as age and gender; intrinsic psychological factors, such as perceived value and usability; and external environmental factors, such as government support and psychological expectations. For those questions, we studied the usage of quality traceability system for navel oranges in the southern GanZhou city (GanNan), in which the navel oranges have national reputation. With the Technology Acceptance Model (TAM) as the pivot, the study delves into the following three key issues: (1) how to construct an analysis model of influencing factors and mechanisms of continuous usage behavior regarding the agricultural product quality traceability system; (2) how to combine the Expectation Confirmation Model (ECM) theory to construct an integrated TAM model of usage behavior towards the agricultural product quality traceability system; (3) how to explore the characteristics variables of usage behavior towards the agricultural product quality traceability system, i.e. intrinsic psychological factors and external environmental factors that may affect their behavior. Partial Least Squares (PLS) analysis method is adoptedto analyze the survey data of 197 producers who have been using the quality traceability system for two years in GanNan navel orange production area.

Compared to existing literature, our contribution is to made new extensions in threemajor aspects: the model selection,the setting of research variables, and the selection of data collection.

In terms of model construction, this study retains two variables from the Technology Acceptance Model (TAM), namely perceived usefulness and intention to use, and two variables from the Expectation-Confirmation Model (ECM), namely confirmation expectation and satisfaction. These are integrated into the TAM-ECM integrated model. The study applies this new integrated theoretical model to empirically test the factors influencing the continuous usage behavior of producers in agricultural product quality traceability systems.

Regarding to research variables, firstly, the study simplifies external environmental variables by including only transaction cost variables. It specifically examines the negative impact of transaction costs on quality traceability systems from the aspects of information costs, communication costs, and supervision costs. Secondly, the study innovates in the configuration of individual characteristic variables by adding the variable of usage habits, to study the interaction between satisfaction and user's continuous usage behavior. Prior literature has relatively underexplored this user behavior characteristic, and this study contributes by addressing this gap. Finally, through communication with producer users, the study identifies that the key reason for users to adopt the quality traceability system is the desire for their products to be accepted by consumers at higher prices, leading to greater profits. They perceive the use of the system as valuable. Consequently, this study transforms the perceived usefulness variable from the TAM model into the perceived value variable.

In terms of data collection and selection, the study focuses on producers who have been using the GanNan navel orange quality traceability system for duration of 2 years, ensuring the quality of the empirical research results.

Our tests show the TAM-ECM integrated model has a good explanatory power. The most important conclusion of our research is that satisfaction can not only directly promote the continuous use of the quality traceability system but also further drive the willingness to continue using it through the mediating variable of usage habits. This conclusion is consistent with actual situations, indicating that user habits will create positive feedback on their willingness to keep using the system.

The structure of the remaining part of this paper is arranged as follows: Part 2 is literature review. Part 3 describes research hypotheses, and the construction of the TAM-ECM integration model. Part 4 conducts empirical tests, including descriptive statistics, reliability and validity tests, as well as structural equation modeling. The final part provides the summary.

## Literature review

### Agricultural product quality traceability systems

Agricultural product quality traceability systems refer to the use of information technology to attach a "dedicated" label to agricultural products. This label contains information about the cultivation, processing, packaging, logistics, and other aspects of the agricultural product. By querying the label, one can trace the handlers at each stage, providing a reliable basis for the government to address food safety and quality supervision issues.

For the quality traceability system for agricultural products, producers are the ultimate users of the system. The perceived ease of use, expected benefits, and usage habits of users will affect their willingness to use and usage behavior^4^. The expected benefits of users reflect their satisfaction with the quality traceability system for agricultural products. When the difference between investment and expected benefits is too large, producers are likely to stop using the system. Therefore, studying the influencing factors and mechanisms of users' continuous usage behavior is of great practical significance in promoting their active participation in using the traceability system for agricultural products quality.

### Transaction cost theory

According to the Transaction Cost Theory, the choice of agricultural producers and users for a quality traceability system is essentially a contract between producers and the government in a specific environment. The promotion of the quality traceability system by the government and the technological choices made by users involve information asymmetry. In situations of information asymmetry, users face the impact of information costs, communication costs, and supervision costs in their decision-making process regarding the use of agricultural product quality traceability systems due to issues such as knowledge barriers and trust in the government.

Research on user behavior is one of the most widely applied areas of transaction cost theory. In the field of user behavior, transaction cost research primarily involves four major variables: perceived value, perceived usability, user satisfaction, and user intention to use. The impact of transaction costs on these variables includes direct/mediating effects and positive/negative/insignificant effects. According to studies by Yuen et al.^5^ and Liang et al.^6^, a reduction in transaction costs can lead to an increase in perceived value, thereby enhancing user intention to use. Additionally, the asset specificity within transaction costs has a positive impact on perceived usability. In Li et al.'s^7^ research, transaction costs can mediate user satisfaction by influencing the perceived achievement of goals. Finally, a decrease in transaction costs can strengthen users' continuous intention to use, as evidenced by studies by Yuen et al.^5^, which yielded similar results.

### Technology acceptance model

The Technology Acceptance Model (TAM), first proposed by Davis^8^, focuses on analyzing the influence of perceived ease of use and perceived usefulness on users' acceptance and usage behavior of technology. The main variables include perceived cost and perceived risk. With the development of new technologies, such as electronic gaming consoles, VR devices, and especially the emergence of artificial intelligence technology, the TAM model continues to iterate, optimizing its variables and integrating with other theories. This makes it more applicable for explaining users' intentions and behaviors in adopting information technology. For example, Nan et al.^9^ combines the Coolness factor with TAM theory to study the usage intention of video gaming consoles.Many scholars have used TAM and its extended models as a theoretical basis to study agricultural information technology acceptance behavior (Table 1).

**Table 1:** Literature on the application of TAM model in agricultural information technology.

Author (Time)	Research content	Theoretical models	Variable measurement
Matthias et al. 2011^10^	Food tracking and tracing system	TAM model	Perception cost, external pressure
AlTal 2012^11^	Food traceability system	TAM model	Perceived risk
Pappa et al. 2018^12^	Use of traceable systems in agricultural supply chains	TAM2-TPBintegrated model	Perception cost
Feng and Li 2019^13^	Information on vegetable facilities for farmers	TAM model	Perception cost, perceived value
Li et al. 2020^3^	Farmer participation in vegetable traceability system	TTF model	External environment, system characteristics
Dai and Cheng 2022^14^	Farmer continued use of green planting technology	TAM model	Perceived risk

### Expectation confirmation theory

Expectation Confirmation Theory (ECT), founded by Oliver^15^, is a theory used to explain user satisfaction. ECT quantifies consumer satisfaction by comparing their expectations of a product before purchasing it with its performance during use. If the expectations exceed the initial expectations, then the customer is satisfied; if the expectations are lower than the initial expectations, then the customer is dissatisfied^16^.

Based on ECT theory, Bhattacherjee^17^ constructed the Expectation Confirmation Model (ECM). The ECM model mainly includes several constructs such as perceived value, expectation confirmation, satisfaction, and continued usage intention. Expectation confirmation refers to the difference between a user's initial expectations and actual perceptions. The higher the degree of expectation confirmation, the more valuable the system is perceived by the user, which then enhances their satisfaction with the information system and further generates continued usage intention^18^.

In the existing literature that uses the ECM model, most scholars use it to study the continued usage intention and behavior of information system users (Table 2). The main variable, expected confirmation level, is a direct influencing factor on users' continued usage behavior and satisfaction. Therefore, the ECM model is suitable for the objects of this study.

**Table 2:** Literature of ECM model in continuous use behavior.

Author (Time)	Research content	Theoretical models	Variable measurement
Yin and Li 2017^19^	Continuous usage intention of apps	ECT theory	Degree of expected confirmation, perceived usefulness
Zhang et al. 2020^20^	Continuous usage intention of social media apps	ECM model	Degree of expected confirmation, satisfaction
Liuet al. 2022^21^	Continuous vaccination intention	ECM model	Degree of expected confirmation, perceived value
Liet al. 2022^7^	Continuous usage intention of online learning	ECM model	Degree of expected confirmation, perceived usefulness, satisfaction
Leouand Wang 2023^22^	Prediction of tourists' intention to revisit	ECM model and TPB model	Degree of expected confirmation, satisfaction

Research on the use of technology in information management has found that TAM and ECM models are complementary to each other. Premkumar and Bhattacherjee^23^ argued that TAM is a cross-sectional model in theory because it can predict the use of an information system based on users' perceptions at any given point in time, while ECM is a longitudinal (process) model in which usage expectations prior to usage and time usage structures (such as performance, uncertainty, and satisfaction) are separated over time, and these structures jointly affect the post-usage of the information system. Therefore, constructing a TAM-ECM integrated model can serve as a theoretical basis for studying the continuous use behavior of producer users in agricultural product quality tracing systems.

Currently, most research on the continuous use intention and behavior of agricultural information systems users chooses to combine the ECM model with other usage behavior explanatory models such as TAM and IS success. The research process has always revolved around the setting of variables and can be roughly divided into three types:

The first is the setting of self-characteristic variables of the research object. The differences in self-characteristics of producer users will have different impacts on the continuous use intention and behavior of the quality traceability system. For example, the age, gender, and education level of producers do not significantly affect their participation in using quality traceability systems, while income and planting scale do have significant effects^2,24^, and some do not^25^. Therefore, further exploration is needed on the impact of producer characteristics on the use intention and behavior of traceability systems.

The second is the setting of internal psychological factor variables. The variables studied in existing literature for internal psychological factors include perceived ease of use, perceived value, expectation confirmation, perceived risk, and perceived cost. Currently, most scholars mainly start from the positive factors that influence the continuous use intention of quality traceability systems and explore the mediating effect of perceived ease of use and perceived value on continuous use intention^2,3,9,24,26–28^. Only a few scholars consider negative influencing factors such as perceived cost and perceived risk^12,26,29,30^. Among them, Wang and Chen^29^ clearly divided the influencing factors into positive and negative aspects in their research on the continuous use intention of new professional farmer information service platforms. They found that perceived value, perceived ease of use, and expectation confirmation have a significant positive impact on continuous use intention, while perceived risk and perceived cost have a significant negative impact. This not only verifies the hypothesis but also is consistent with the advocacy of most existing researchers.

The third is the setting of external environmental factor variables. When using basic TAM, ECM, and other models, many scholars set external environmental variables as government support, technical training, or peer influence^2,3,12,24^. Existing literature generally believes that the government is a key force in motivating producer users to continuously use quality traceability systems. Increasing the frequency of quality inspections by the government will increase the willingness of producers to use traceable systems^31^. On the other hand, many scholars have conducted empirical research on the factors affecting the continuous use intention of quality traceability systems in all aspects of information platforms. The research variables include system quality, information quality, and service quality, etc. The results show that improving system quality and information quality have an important impact on the continuous use intention of quality traceability systems^26,27,29,32–34^. Therefore, the setting of external variables can be further expanded.

In summary, in the current research on the factors influencing the willingness and behavior of using agricultural product quality traceability systems, most of the research hypotheses have been verified and the theoretical models have achieved good explanatory power. This has important theoretical value and practical significance for the study of user behavior in quality traceability systems. However, existing research often produces conflicting results when studying the same variables, possibly due to differences in the setting of research variables, model integration and expansion, or the selection of research subjects. We believe that research in this field needs to be improved in the following aspects: First, in addition to the characteristic variables of the research object itself, behavioral-related characteristics such as usage frequency and habits should also be included as research variables; Secondly, innovative external environmental variable settings are needed, such as including transaction costs as external variables to explore their impact on the willingness and behavior of continuous use of quality traceability systems; Thirdly, the selected research object sample needs to better reflect the general situation of producers and users.

## Research hypothesis and theoretical model

### Research hypothesis

#### Transaction cost, expectation confirmation degree and perceived value

Liang et al.^35^ found on consumer adoption of sharing platforms that reducing transaction costs is beneficial for enhancing their perceived value of the sharing platform. Yuen et al.^5^, in a study on customers' willingness to use smart lockers, also concluded that transaction costs have a negative impact on perceived value. Dai and Liu^36^ combined ECM theory with social presence and used structural equation modeling to analyze 308 survey data collected, finding that expected confirmation degree has a positive impact on perceived value. Yin and Li^19^ conducted a successful theoretical study on health apps by integrating ETC and IS, and the results showed that increasing users' expected confirmation degree of health apps can increase their perceived value of the app. Therefore, we propose the following two hypotheses:

##### H1:

Reducing transaction costs is beneficial for producer-users to enhance their perceived value.

##### H2:

Increasing producer-users' expected confirmation degree is beneficial for improving their perceived value.

#### Transaction cost, expectation confirmation degree and satisfaction

Gorla and Somers^37^ found that reducing transaction costs can increase user satisfaction when assessing the impact of IT outsourcing on information system success. Bhattacherjee^17^ constructed an ECM model and discovered that the degree of expected confirmation can influence users' willingness to continue using a system by affecting their satisfaction. Based on the ECM theory and TAM model, Zhu^38^ used structural equation modeling to validate hypotheses using 589 valid questionnaire data, and found that the degree of expected confirmation has a direct and positive significant impact on perceived usefulness and satisfaction. Additionally, Liu et al.^18^, and Guan^39^ also believe that increasing the degree of expected confirmation can promote user satisfaction and perceived value. Therefore, we propose the following two hypotheses:

##### H3:

Reducing transaction costs is beneficial for producer users to improve satisfaction.

##### H4:

Improving the degree of expected confirmation for producer users is beneficial for increasing their satisfaction.

When studying the O2O online and offline hybrid business model, Hsu and Lin^40^ found that reducing transaction costs enhances customers' willingness to use. Hansen et al.^41^ believed that the perceived value of software transactions would increase users' willingness to use, and there is a positive relationship between the two. Natarajan et al.^42^ discovered in their research that perceived value has a significant positive impact on usage intention. Based on the TAM model, the theory of planned behavior, and the ECM model, Zhou et al.^43^ used empirical testing methods to show that improving perceived usefulness benefits user's continuous intentions to use, using ten universities in Nanjing as an example. Nan et al.^28^ found a positive correlation between user satisfaction and loyalty based on the Coolness factor analysis. Lu et al.^44^ conducted an empirical study on user's continuous intention to use through the TAM-ECM integrated model, which showed that increasing satisfaction was beneficial for enhancing users' continuous intention to use. Wang^45^ found, based on the ECM theory that the higher user satisfaction level leads to stronger intentions of continued participation. Therefore, we propose three hypotheses as follows:

##### H5:

Reducing transaction costs for producer-users is beneficial for enhancing their continuous intention to use.

##### H6:

Improving the perceived value for producer-users is beneficial for enhancing their continuous intention to use.

##### H7:

Improving the satisfaction level for producer-users is beneficial for enhancing their continuous intention to use.

#### Satisfaction, usage habits and willingness to continue using

There is a mutual influence between satisfaction, usage habits, and users' continuous use. In the study of user's continuous use willingness, Liu et al.^46^ found that the improvement of user satisfaction has a significant impact on users' willingness to continue using information technology or systems. Chen et al.^47^ constructed an integration model of TAM-IS for information system user adoption, taking usage habits as a key factor, studying the impact and mechanism of satisfaction and usage habits on users' continuous use behavior, and using usage habits as a mediator to positively affect satisfaction and users' continuous use willingness and behavior. Therefore, we propose the following two hypotheses:

##### H8:

The improvement of producer users' satisfaction is conducive to the improvement of their usage habits.

##### H9:

The improvement of producer users' usage habits is conducive to the improvement of their willingness to continue using.

#### Continuous use intention and continuous use behavior

Liu et al.^18^ proposed the ECM for Continued Use, which suggests that an increase in users' continuous using intention a video website leads to an increase in their actual continued usage behavior. Wu et al.^48^ further argue that the continuous using intention is a core mediator concept in the ECM and factors such as satisfaction and perceived usefulness influence continued usage behavior through this mediator variable. Therefore, the following hypothesis is proposed:

##### H10:

An increase in producers' continuous using intention is favorable for their actual continued usage behavior.

#### The mediating role of perceived value

The above assumptions mention that "the degree of user's expected confirmation and the government's support for the traceability system of agricultural products are beneficial to the increase of perceived value" and "the perceived value of users is beneficial to increasing the willingness of producers and users to continue using the traceability system of agricultural product quality". It can be considered that perceived value plays an intermediary role in the impact of expected confirmation level and transaction cost on the willingness of users to continue using the system. Therefore, we propose the following mediation hypothesis:

##### H11:

The degree of expected confirmation enhances the willingness to continue using through perceived value, and perceived value has a mediating effect.

#### The mediating role of satisfaction

The above assumptions mention that "the degree of user's expected confirmation and the government's support for the traceability system of agricultural products are beneficial to the increase of perceived value" and "the perceived value of users is beneficial to increasing the willingness of producers and users to continue using the traceability system of agricultural product quality". It can be considered that perceived value plays an intermediary role in the impact of expected confirmation level and transaction cost on the willingness of users to continue using the system. Therefore, we propose the following mediation hypothesis:

##### H12:

The degree of expected confirmation enhances the willingness to continue using through perceived value, and perceived value has a mediating effect.

#### The mediating effect of usage habits

The above assumptions mention that "user satisfaction with the system promotes the habit of using the system (UH)" and "as the usage habit increases, the willingness to continue using the system (CUI) will also increase." It can be considered that usage habits act as a mediator variable, producing indirect promotion effects between satisfaction and user continued usage behavior. Therefore, we propose the following mediation hypothesis:

##### H13:

Satisfaction enhances its willingness to continue using the system through the usage habits, and usage habits have a mediating effect.

### Model construction

**Figure 1:**
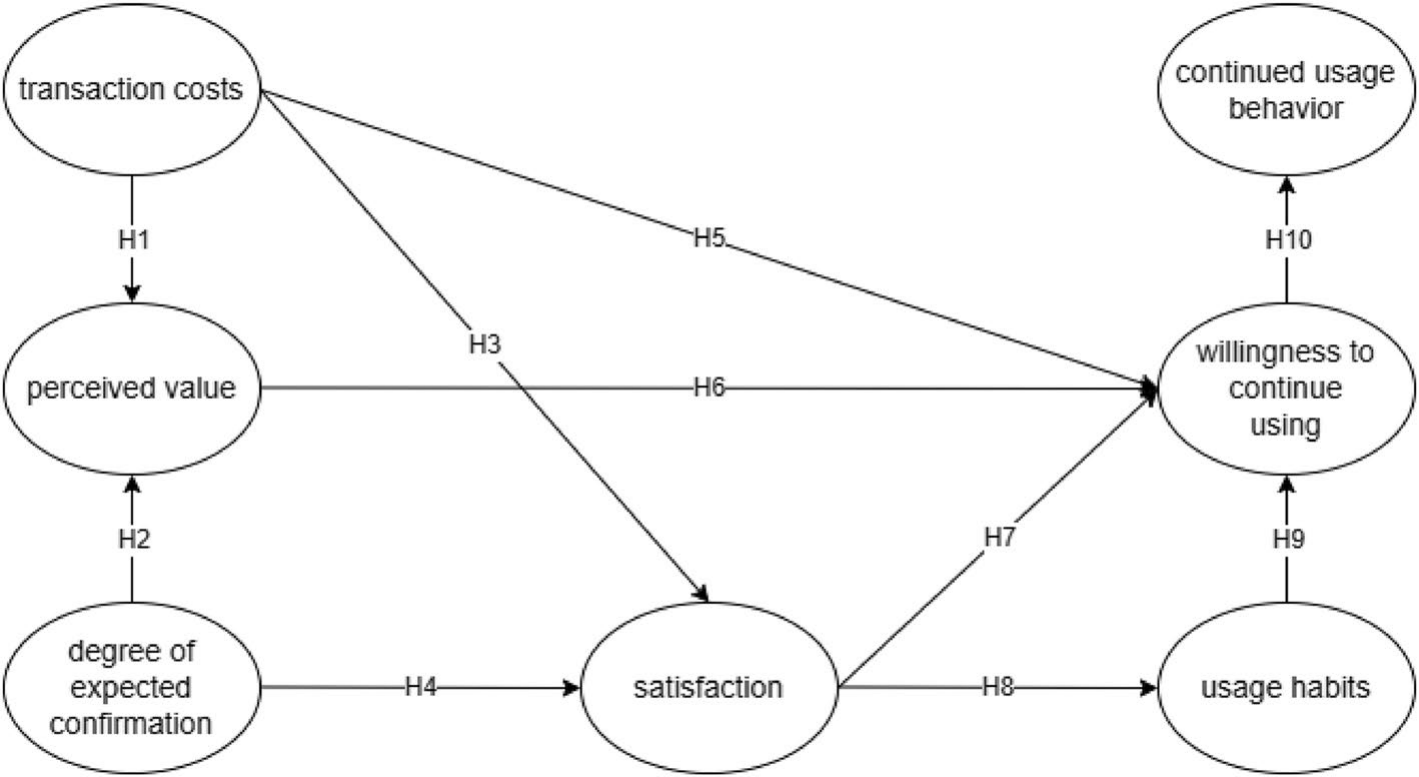
Integrative theoretical model of TAM-ECM.

Based on TAM model and ECM model, we build a research model of producer users' willingness to continue using TAM-ECM on the quality traceability system of Jiangxi navel orange. The hypothesis in the TAM-ECM model is shown in Fig. 1.

### Variables

The continuous use intention and use behavior of the agricultural product quality traceability system are affected by the cognitive belief, expectation degree and external environment of the producers and users. Users' perceived ease of use, expected degree of confirmation and transaction cost are taken as dependent variables, perceived value, satisfaction and usage habits are taken as mediating variables, and continuous use intention and continuous use behavior are taken as independent variables. The specific variable measures are described in Table 3.

**Table 3:** Description of TAM-ECM integration model variables.

Class	Latent variable	Observed variable	Document source
Antecedent variable	Perceived ease of use	I learned the use of the quality traceability system very easily(PEOU1)	Davis 1986^8^; Davis et al. 1989^49^; Venkatesh and Davis 2000^50^; Venkateshs et al. 2003^51^; Hsieh et al. 2008^52^; Venkatesh and Bala 2008^53^
		It doesn't take me much time to operate the quality traceability system (PEOU2)	
		I find it easy to operate the quality traceability system (PEOU3)	
		Overall, I find it easy for me to use the quality traceability system (PEOU4)	
	expectation degree of confirmation	After using the quality traceability system, I found that the harvest exceeded my expectations (EDOC1)	Oliver 1980^15^; Bhattacherjee 2001^17^
		After using the quality traceability system, I found the quality of the features exceeded my expectations (EDOC2)	
		After using the quality traceability system, I found that the number of features exceeded my expectations (EDOC3)	
		In general, my expectations for the quality traceability system were met after use (EDOC4)	
	transaction costs	I have easy access to information about participating in the quality traceability system (TC1)	Hobbs 1997^54^
		It was easy for me to communicate with the technical staff in the process of using the quality traceability system (TC2)	
		The government will supervise my use of the quality traceability system and evaluate the effect (TC3)	
Mediating variable	perceived value	The quality traceability system is used to ensure the quality of navel oranges (PV1)	Davis 1986^8^; Davis et al. 1989^49^; Venkatesh and Davis 2000^50^; Venkatesh et al. 2003^51^; Srite and Karahanna 2006^55^; Venkatesh and Bala 2008^53^
		Use a quality traceability system to standardize orchard management (PV2)	
		The use of a quality traceability system has led to higher prices for navel oranges (PV3)	
		The use of a quality traceability system leads to higher income for orchards (PV4)	
		Overall, it was valuable for me to use the quality traceability system (PV5)	
	satisfaction	I was satisfied with the training and service of the quality traceability system (SAT1)	Oliver 1980^15^; Smithson and Hirschheim 1998^56^; Bhattacherjee 2001^17^; DeLone and McLean 2003^57^
		I am satisfied with my experience with the quality traceability system (SAT2)	
		I am satisfied with the use of the quality traceability system (SAT3)	
		Overall, I am satisfied with the quality traceability system (SAT4)	
	usage habits	Using a quality traceability system has become a habit for me (UH1)	Chenetal 2014^47^
		Using a quality traceability system is natural to me (UH2)	
		As I continued orchard management, a quality traceability system was an obvious choice for me (UH3)	
Result variable	continuous using intention	I would like to continue using the quality traceability system (CUI1)	Davis 1986^8^; Davis et al. 1989^49^; Venkatesh and Davis 1996^58^; Venkatesh and Davis 2000^50^; Venkateshs et al. 2003^51^
		I will continue to use the quality traceability system (CUI2)	
		I would recommend the quality traceability system to other fruit farmers (CUI3)	
	continuous use behavior	I will often use the quality traceability system (CUB1)	Davis 1986^8^; Davis et al. 1989^49^; Igbaria et al. 1995b^59^; Venkateshs et al. 2003^51^; Burton Jones and Straub. 2006^60^; Karahanna et al. 2006^61^; Devaraj et al. 2008^62^
		I will make extensive use of the quality traceability system (CUB2)	
		I would recommend quality traceability to other farmers (CUB3)	

We focuses on the TAM-ECM integrated model and selects Expectation Degree of Confirmation (EDOC), Perceived Ease of Use (PEOU), Perceived Value (PV), Transaction Cost (TC), Satisfaction (SAT), Usage Habits (UH), Continuous Use Intention (CUI), and Continuous Use Behavior (CUB) as the research dimensions to explore the factors and mechanisms that affect the continuous use intention and behavior of producer users in the context of the technical background of the quality traceability system for GanNan navel oranges.

## Data and empirical tests

### Data sources

The data used in this study were collected from 18 counties in the southern Ganzhou City, Jiangxi Province from July 20 to August 20, 2019.The subjects of this survey are loyal users who have completely used the quality traceability system of Jiangxi navel orange for more than two years. All of them have participated in the training of the quality traceability system of Jiangxi navel Orange organized by Ganzhou Fruit Industry Bureau, and the effective sample number is 197.

#### Questionnaire design

The survey questionnaire is designed to target users' demographic information, business scale, income, degree of confirmation of expectations, user awareness, transaction costs, usage habits, and the willingness to continue using the GanNan navel orange quality traceability system. Each construct item uses a Likert seven-point scale. The questionnaire design still follows the four-step process of "initial measurement item design based on reference literature" → "small-scale interviews for revision" → "small-scale reliability and validity testing" → "revision and formation of final questionnaire."

## Sample characteristics

This survey adopted the method of combining questionnaire survey and typical user interview, and 197 valid samples were finally collected. Five questions were set in the questionnaire about users' basic information, including gender, age, education level, planting area and income. Table 4 lists the details.

**Table 4:** Basic information of the respondents.

Variable	Variable interpretation	Frequency/person	Proportion /%
Gender	Male	140	71.1
	Female	57	28.9
Age/year	< 30	20	10.2
	30 ~ 39	83	42.1
	40 ~ 49	84	42.6
	≧50	10	5.1
Educational level	Primary school	1	0.5
	Junior high school	10	5.1
	Senior high school	95	48.2
	College degree or above	91	46.2
Planting area (mu)	< 10	2	1.0
	10 ~ 29	6	3.0
	30 ~ 49	60	30.5
	50 ~ 199	91	46.2
	≧200	38	19.3
Income (￥10,000)	< 10	10	5.1
	10 ~ 29	26	13.2
	30 ~ 50	57	28.9
	50 ~ 100	61	31.0
	> 100	43	21.8

### Empirical analysis

#### Analysis of measurement model

##### Questionnaire reliability test

As can be seen from Table 5, the α coefficients of the eight dimensions of expected confirmation degree, transaction cost, perceived ease of use, perceived value, satisfaction degree, usage habit, continuous using intention and behavior to continue using are 0.829, 0.757, 0.860, 0.758, 0.841, 0.874, 0.757 and 0.775, respectively, and all above 0.7. Therefore, the reliability of the questionnaire sample data is good.

**Table 5:** Questionnaire reliability.

Variable	Cronbach’s α
Expectation degree of confirmation	0.829
Transaction costs	0.766
Perceived value	0.758
Satisfaction	0.841
Usage habits	0.874
Continuous using intention	0.757
Continuous use behavior	0.775

##### Convergence validity test

Table 6 shows that the standard factor loading of all measurement items is greater than 0.6, the synthesis reliability is greater than 0.8, and the average variance sampling is higher than 0.5. It can be considered that the model is acceptable and has good convergence validity.

**Table 6:** Convergent validity.

Variable	Measure term	Standard factor load	CR	AVE
Expectation degree of confirmation	EDOC1	0.813	0.886	0.661
	EDOC2	0.787		
	EDOC3	0.829		
	EDOC4	0.822		
Transaction costs	TC1	0.854	0.864	0.681
	TC2	0.713		
	TC3	0.898		
Perceived value	PV1	0.737	0.837	0.509
	PV2	0.712		
	PV3	0.642		
	PV4	0.629		
	PV5	0.829		
Satisfaction	SAT1	0.832	0.893	0.677
	SAT2	0.770		
	SAT3	0.826		
	SAT4	0.862		
Usage habits	UH1	0.891	0.922	0.798
	UH2	0.896		
	UH3	0.893		
Continuous using intention	CUI1	0.877	0.861	0.675
	CUI2	0.826		
	CUI3	0.757		
Continuous use behavior	CUB1	0.825	0.870	0.690
	CUB2	0.846		
	CUB3	0.821		

##### Discrimination validity test

The method proposed by Fornell and Larcker^63^ is adopted for discrimination validity analysis. Whether the square root of AVE is higher than the correlation coefficient of two factors' perspectives is used to judge whether there is discrimination validity. Table 7 shows the correlation coefficients of the eight dimensions and the square root of AVE (i.e., the value on the diagonal). The square root of AVE is greater than the correlation coefficient between facets, which means that the model has good discriminative validity.

**Table 7:** Discriminant validity.

	Expectation degree of confirmation	Transaction costs	Perceived value	Satisfaction	Usage habits	Continuous using intention	Continuous use behavior
Expectation degree of confirmation	0.813						
Transaction costs	0.527	0.825					
Perceived value	0.607	0.498	0.713				
Satisfaction	0.697	0.609	0.631	0.823			
Usage habits	0.570	0.417	0.441	0.685	0.893		
Continuous using intention	0.620	0.632	0.606	0.680	0.582	0.822	
Continuous use behavior	0.518	0.487	0.560	0.504	0.566	0.781	0.831

The diagonal is the square root of AVE.

#### Structural model analysis

##### Direct effect test

Table 8 shows the direct effect test results of partial least squares model (PLS) analysis. The number of Bootstrapping times was 5000.

**Table 8:** Hypothesis test results of main variables.

Hypothesis	Hypothesis relation	Path coefficient	p-value	Confidence interval	Hypotheses test
H1	Transaction costs → perceived value	0.247	0.000	0.121 ~ 0.366	Supported
H2	Expectation degree of confirmation → perceived value	0.477	0.000	0.359 ~ 0.580	Supported
H3	Transaction costs → satisfaction	0.334	0.000	0.184 ~ 0.471	Supported
H4	Expectation degree of confirmation → satisfaction	0.521	0.000	0.417 ~ 0.626	Supported
H5	Transaction costs → continuous using intention	0.304	0.000	0.185 ~ 0.424	Supported
H6	Perceived value → continuous using intention	0.233	0.000	0.116 ~ 0.342	Supported
H7	Satisfaction → continuous using intention	0.200	0.028	0.018 ~ 0.377	Supported
H8	Satisfaction → usage habits	0.685	0.000	0.577 ~ 0.768	Supported
H9	Usage habits → continuous using intention	0.216	0.004	0.074 ~ 0.366	Supported
H10	Continuous using intention → continuous use behavior	0.781	0.000	0.715 ~ 0.832	Supported

The confidence level is 95%.

The effect of transaction cost on perceived value is significant at the level of 0.000, the path coefficient is 0.247, and the confidence interval (0.121 ~ 0.366) does not include 0, that is, H1 passes the test, and the hypothesis is supported.

Expected confirmation has a significant effect on perceived value and satisfaction at the level of 0.000. The path coefficients are 0.477 and 0.521, and the confidence intervals (0.359–0.580) and (0.417–0.626) do not include 0, indicating that H2 and H4 pass the test and the hypothesis is supported.

Transaction cost has a significant effect on satisfaction at the level of 0.000, the path coefficient is 0.334, the confidence interval (0.184 ~ 0.471) does not include 0, that is, H3 passes the test, and the hypothesis is supported.

The transaction cost has a significant effect on the willingness to continue using at the level of 0.000, the path coefficient is 0.304, the confidence interval (0.185–0.424) does not include 0, that is, H5 passes the test, and the hypothesis is supported.

The perceived value has a significant effect on the continuous using intention at the level of 0.000, the path coefficient is 0.233, and the confidence interval (0.116 ~ 0.342) does not include 0, indicating that H6 has passed the test and the hypothesis is supported.

Satisfaction has a significant effect on the continuous using intention at the level of 0.05, the path coefficient is 0.200, and the confidence interval (0.018 ~ 0.377) does not include 0, indicating that H7 passes the test and the hypothesis is supported.

Satisfaction has a significant effect on usage habits at the level of 0.000, the path coefficient is 0.685, and the confidence interval (0.577 ~ 0.768) does not include 0, indicating that H8 has passed the test and the hypothesis is supported.

The relationship between use habit and continuous use intention was significant at the level of 0.01, and the path coefficient was 0. 216. The confidence interval (0.074 ~ 0.366) does not include 0, indicating that H9 passes the test and the hypothesis is supported.

The willingness to continue use has a significant effect on the behavior of continuous use at the level of 0.000, the path coefficient is 0.781, and the confidence interval (0.715–0.832) does not include 0, indicating that H10 has passed the test and the hypothesis is supported.

**Figure 2:**
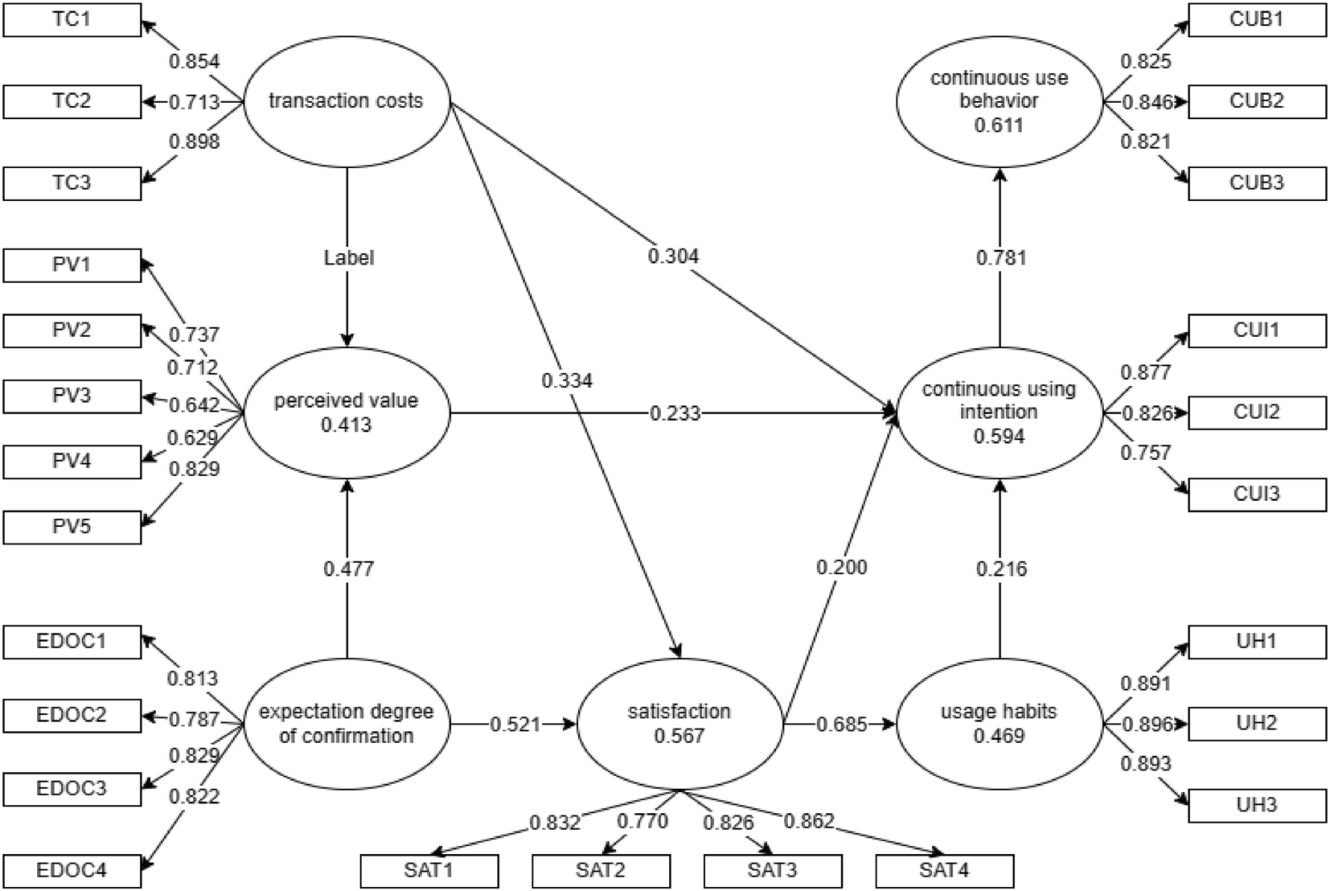
Path map of TAM-ECM integration model.

Figure 2 is a path diagram of the TAM-ECM-based continuous usage intention model of the GanNan navel orange quality traceability system for producer users under transaction costs, which reflects the size of the path relationship between direct assumptions and the explanatory power of variables.

From Fig. 2, it can be seen that when the expected confirmation level increases by 1 unit, the perceived value of the user will increase by 0.477 units; when the expected confirmation level increases by 1 unit, the satisfaction of the user will increase by 0.521 units; when the transaction cost decreases by 1 unit, the perceived value of the user will increase by 0.247 units; when the transaction cost decreases by 1 unit, the satisfaction of the user will increase by 0.334 units; when the perceived value increases by 1 unit, the user's willingness to continue using will increase by 0.233 units; when satisfaction increases by 1 unit, the user's usage habit will increase by 0.685 units; when satisfaction increases by 1 unit, the user's willingness to continue using will increase by 0.2 units; when usage habits increase by 1 unit, the user's willingness to continue using will increase by 0.216 units; when willingness to continue using increases by 1 unit, the user's continuous use behavior will increase by 0.781 units.

The expected confirmation level and transaction cost together explain 41.3% of the variance in perceived value, among which the impact of expected confirmation level on perceived value is greater than the impact of transaction cost on perceived value; transaction cost and expected confirmation level together explain 56.7% of the variance in satisfaction, with the impact of expected confirmation level being greater than that of transaction cost; usage habits, perceived value, satisfaction, and transaction cost together explain 59.4% of the variance in willingness to continue using, with the impact ranked in descending order from transaction cost, perceived value, usage habits, to satisfaction; satisfaction alone explains 46.9% of the variance in usage habits; willingness to continue using alone explains 61.1% of the variance in continuous use behaviors.

##### Intermediate effect test

The impact and significance of the mediator assumption can be seen from Table 9. The specific relationships are as follows:

**Table 9:** Intermediate effect test results.

Hypothesis	Hypothesis relation	Total effect test	p-value	Confidence interval	Effect size	Hypotheses test
H11	Expectation degree of confirmation → perceived value → continuous using intention	Pass	0.001	0.052 ~ 0.178	0.111	Supported
H12	Expectation degree of confirmation → satisfaction → continuous using intention	Pass	0.035	0.011 ~ 0.205	0.104	Supported
H13	Satisfaction → usage habits → continuous using intention	Pass	0.006	0.052 ~ 0.264	0.148	Supported

The confidence level is 95%.

In the mediating effects of expected confirmation degree → perceived value → willingness to continue using, satisfaction → usage habit → willingness to continue using, P < 0.01, with confidence intervals of (0.052 ~ 0.178) and (0.052 ~ 0.264) respectively, not including 0, indicating that the mediating effects are significant; In the mediating effects of expected confirmation degree → satisfaction → willingness to continue using, P < 0.05, with a confidence interval of (0.0111 ~ 0.205), not including 0, indicating that the mediating effect exists.

The above analysis reflects that all three mediator assumptions, H11, H12, and H13, have passed the test, and perceived value, satisfaction, usage habit, and willingness to continue using play a mediating role in the influence of expected confirmation degree and transaction costs on the continuous use behavior of quality traceability system producer users.

## Discussion and conclusion

We use Partial Least Squares (PLS) analysis to analyze user survey data from 197 users of the GanNan navel orange quality traceability system. Through building a theoretical model integrating Technology Acceptance Model (TAM) and Expectation-Confirmation Model (ECM), this study analyzes the factors affecting producer users' continuous intention and behavior to use the quality traceability system. The reliability test, validity test, measurement model, and structural model analysis tested ten main effect hypotheses and three mediating effect hypotheses. Empirical results show that all ten main effect hypotheses and two mediating effect hypotheses are supported, and the integrated TAM-ECM model can effectively explain producer users' continuous intention and behavior to use the quality traceability system.

Psychological expectation factors of producer users, are crucial factors influencing the satisfaction and continuous usage intention of producer users in quality traceability systems. The improvement in the degree of expectation confirmation can indirectly affect the continuous usage intention of producer users through its impact on perceived value or satisfaction. Enhancing the degree of expectation confirmation can ultimately promote the continuous usage intention of the quality traceability system.

Cognitive belief factors of producer users, specifically perceived value, are intrinsic key factors influencing the continuous usage intention of producer users in quality traceability systems. Perceived value significantly enhances the continuous usage intention of producer users in quality traceability systems. The stronger the belief of producer users that the quality traceability system contributes to their production and sales processes, the more robust their intention to continuously use the system. Therefore, the design of quality traceability systems should consider functionality, and in the promotional process, make producer users perceive the value of the quality traceability system.

Among external factors, transaction costs are crucial factors influencing the satisfaction and perceived value of producer users. Reducing transaction costs helps producer users enhance satisfaction and perceived value in the quality traceability system, indirectly affecting their continuous usage intention. Lowering information costs, communication costs, and supervision costs for farmers using the quality traceability system significantly improves their satisfaction and perceived value of the system, ultimately promoting the continuous usage intention and behavior of producer users.

The usage habits of producer users play an intermediate role in the model of continuous usage behavior in quality traceability system producer users. Reducing transaction costs for producer users in using the quality traceability system and enhancing their satisfaction with the system can influence their continuous usage intention by affecting farmers' usage habits.

Based on the above research conclusions, this study proposes corresponding countermeasures for government departments, fruit industry associations, and producer users to promote the quality traceability system of agricultural products, especially the GanNan Navel Orange quality traceability system, from aspects such as improving user expectation confirmation, enhancing perceived value, and reducing transaction costs.

Government departments are suggested to strengthen the training of producer users in the use of agricultural product quality traceability systems, reducing users' psychological resistance to new things, and developing good and sustainable usage habits. At the same time, using blockchain technology, Internet of Things technology, and other technologies to improve the construction of agricultural product quality traceability system platforms can further reduce transaction costs and enhance users' expectation confirmation.

Fruit industry associations should cooperate with the government in promoting and constructing agricultural product quality traceability systems, informing producer users of their value in food quality and safety assurance, and enhancing new users' expectations for quality traceability systems, especially the GanNan Navel Orange quality traceability system.

With the limitations of this study, future research could explore the correlation between individual characteristic information and the continuous usage behavior of producer users. Additionally, our study did not incorporate element characteristics of different producer users, such as the scale of their farm, income level, and level of organizational socialization, as control variables. Future studies could investigate the impact of these factors on user's continuous usage behavior.

### Ethics declarations

All methods were carried out in accordance with relevant guidelines and regulations. All experimental protocols were approved by the Academic Committee of the School of International Economics and Trade, Jiangxi University of Finance and Economics. Informed consent was obtained from all subjects and/or their legal guardian(s).

## Data Availability

The data that support the findings of this study are available fromthe corresponding author upon reasonable request.
